# PHYSICAL ACTIVITY TRENDS IN A NATIONWIDE MHEALTH PROGRAM: A POPULATION-BASED COHORT STUDY

**DOI:** 10.1093/geroni/igac059.982

**Published:** 2022-12-20

**Authors:** Gregory Ang, Sarah Edney, Chuen Seng Tan, Nicole Lim, Jeremy Tan, Falk Riemenschneider, Cynthia Chen

**Affiliations:** National University of Singapore, Singapore, Singapore; National University of Singapore, Singapore, Singapore; National University of Singapore, Singapore, Singapore; Health Promotion Board, Singapore, Singapore; Health Promotion Board, Singapore, Singapore; National University of Singapore, Singapore, Singapore; National University of Singapore, Singapore, Singapore

## Abstract

Physical inactivity is a global public health challenge, leading to an increase in chronic diseases. Effective large-scale physical activity interventions are needed. We examined the effectiveness of a nationwide mHealth intervention in Singapore, National Steps Challenge Season 3 (NSC3). NSC3 included gain-framed financial incentives for reaching pre-defined daily step targets, nudging via reminders and real-time feedback on physical activity levels. Our study includes 411,528 participants, with 53,371 (13.0%) participants aged 59 and above. Regression discontinuity design examined changes to daily step counts prior to and during NSC3. NSC3 was associated with an overall mean increase of 1437 steps per day (95% CI: 1408 to 1467). Participants in older age groups were associated with larger mean step increases. Females aged between 59 to 68 and 69 and above were associated with average increases of 1640 steps per day (95% CI: 1510 to 1770) and 2300 steps per day (95% CI: 2050 to 2550), respectively. Males in the same age groups were associated with average increases of 1360 steps per day (95% CI: 1200 to 1520) and 1640 steps per day (95% CI: 1370 to 1900), respectively. We provide real-world evidence that suggests NSC3 improved participants’ step counts, with a larger increase among older adults. As older adults have a higher risk of chronic diseases, lower physical inactivity has potential health benefits such as reducing the incidence of hypertension and improving cardiovascular health biomarkers. While results are promising, further investigations are necessary to ensure sustained engagement among older adults.

